# A Comprehensive Review of the Phytochemical, Pharmacological, and Toxicological Properties of *Tribulus terrestris* L.

**DOI:** 10.3390/biom10050752

**Published:** 2020-05-12

**Authors:** Ruxandra Ștefănescu, Amelia Tero-Vescan, Ancuța Negroiu, Elena Aurică, Camil-Eugen Vari

**Affiliations:** 1 Department of Pharmacognosy and Phytotherapy, Faculty of Pharmacy, George Emil Palade University of Medicine, Pharmacy, Science, and Technology of Targu Mures, 540139 Targu Mures, Romania; negroiuanca@yahoo.com (A.N.); ele_aurica@yahoo.com (E.A.); 2 Department of Biochemistry, Faculty of Pharmacy, George Emil Palade University of Medicine, Pharmacy, Science, and Technology of Targu Mures, 540139 Targu Mures, Romania; amelia.tero-vescan@umfst.ro; 3 Department of Pharmacology and Clinical Pharmacy, George Emil Palade University of Medicine, Pharmacy, Science, and Technology of Targu Mures, 540139 Targu Mures, Romania; camil.vari@umfst.ro

**Keywords:** *Tribulus terrestris*, phytopharmacology, saponosides

## Abstract

The general spread of *Tribulus terrestris* L. (South Africa, Australia, Europe, and India), the high content of active ingredients (in particular sterol saponins, as well as flavonoids, tannins, terpenoids, phenol carboxylic acids, and alkaloids), and its frequent uses in folk medicine, and as food supplements highlight the importance of evaluating its phytopharmacological properties. There are miscellaneous hypotheses that the species could have a high potential for the prevention and improvement of various human conditions such as infertility, low sexual desire, diabetes, and inflammatory diseases. Worldwide, numerous herbal supplements are commercialized with indications mostly to improve libido, sexual performance in both sexes, and athletic performance. Phytochemical studies have shown great disparities in the content of active substances (in particular the concentration of furostanol and spirostanol saponoside, considered to be the predominant active ingredients related to the therapeutic action). Thus, studies of experimental pharmacology (in vitro studies and animal models in vivo) and clinical pharmacology (efficacy and safety clinical trials) have sometimes led to divergent results; moreover, the presumed pharmacodynamic mechanisms have yet to be confirmed by molecular biology studies. Given the differences observed in the composition, the plant organ used to obtain the extract, the need for selective extraction methods which are targeted at the class of phytocompounds, and the standardization of *T. terrestris* extracts is an absolute necessity. This review aims to highlight the phytochemical, pharmacological, and toxicological properties of *T. terrestris*, with a focus on the contradictory results obtained by the studies conducted worldwide.

## 1. Introduction

*Tribulus terrestris* (TT) is a plant that grows especially in South Africa, Australia, India, and Europe. It is part of the Zygophyllaceae family, a widespread family with 25 genera and about 250 species. TT is a crawling herbal plant that generally grows in arid climates and sandy soils and grows up to one meter high. The name *Tribulus* comes from the Greek name “*tribolos”* which means spike fruit. The fruits are used in traditional Chinese medicine (TCM), in Ayurvedic medicine in India, and traditional medicine in Bulgaria for the treatment of different conditions [1]. In addition, the fruits have monographs in the Japanese Pharmacopoeia, 16th Ed. (2012), Korean Pharmacopoeia, 9th Ed. (2007), Pharmacopoeia of China (2005), and Siddha Pharmacopoeia India, Vol. 1 (2008) (taxonomy validated in http://mpns.kew.org/mpns-portal/). Many compounds with a variety of biological properties and chemical structures have been identified in TT extract, especially steroidal saponins, flavonoids, tannins, terpenoids, polyphenol carboxylic acids, and alkaloids. The composition of TT extract depends on various factors such as the extraction method and whether roots, leaves, or fruits have been used. 

Furthermore, the composition and biological activity of TT depends on growth conditions, including soil quality, but also the harvesting period [2]. As shown by Dinchev et al. [3], the highest content of saponins in the aerial parts was met during the preflowering and flowering periods. However, a correlation could not be found between the geographical and ecological conditions and the chemical composition. Nevertheless, remarkable variations (different concentrations in compounds as well as the absence of some compounds) were noticed between samples collected from the same country [4]. Worldwide, there are many pharmaceutical preparations and herbal supplements that contain extracts standardized in steroidal saponins. These are mainly indicated in libido disorders for both males and females, erectile dysfunction, and abnormal sperm motility, but data from the literature are somewhat controversial regarding the efficacy of TT extracts in such disorders [5]. Increased consumption of TT supplements has also been observed in athletes as they continually seek natural sources for boosting their performance. 

Several reviews have been published in recent years. Table 1 comprises all the reviews related to TT found in the scientific literature.

This review presents the most important phytochemical and pharmacological data with an emphasis on the prominent information related to the chemical composition, pharmacological studies, mechanisms of action, and toxicological data. 

The information on TT was compiled via an electronic search of the following major scientific databases: Science Direct, PubMed, Web of Science, and Scopus, from 2000 to 2020. Whenever the published data were relevant for the present review, the search was extended to 1982 (identification of compounds in different organs and toxicological reports). The query was supplemented by searching the reference lists of papers included in the first selection. The search terms were as follows: “*Tribulus terrestris”* alone or in combination with “chemistry”, “pharmacology”, “effects”, and “toxicity”. For this review, only full-text articles written in the English language were taken into consideration. Unpublished results or grey literature were not included and only pharmacological actions that demonstrated effects both in vitro and in vivo were discussed in the present review.

## 2. Chemical Composition

**Figure 1:**
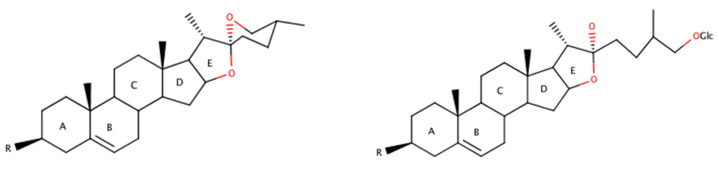
Spirostanol (**left**) and furostanol (**right**) saponins.

TT fruits contain important secondary metabolites such as saponins, polyphenolic compounds, and alkaloids. The steroidal saponins are mainly furostanol and spirostanol type (Figure 1). The furostanol saponins are believed to be biogenetic precursors of the spiro analogs. To date, over 70 different compounds have been identified in TT (Table 2).

Studies have revealed that the composition is strictly linked with the origin of the plant, and hence with climatic conditions.

**Figure 2:**
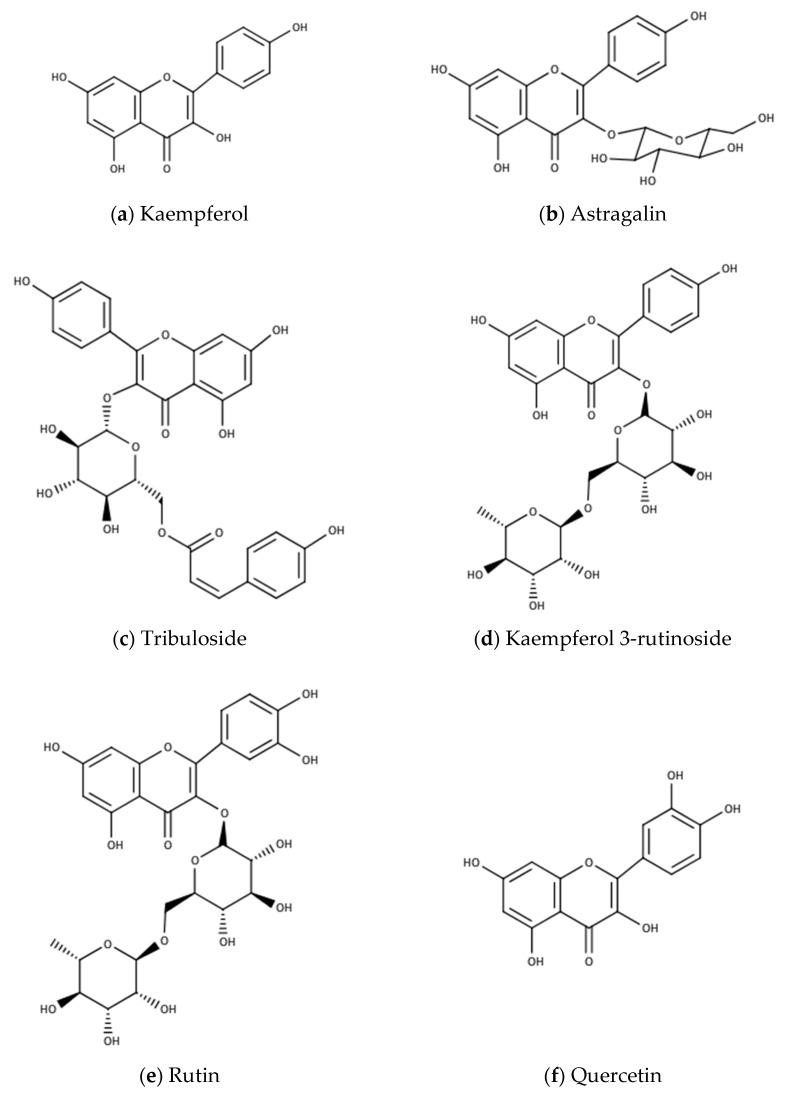

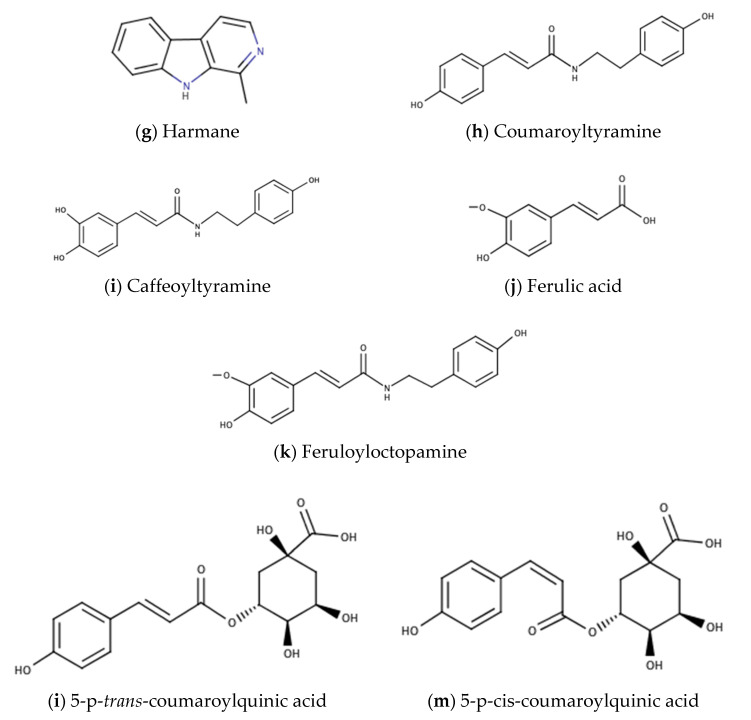
The most common compounds found in TT extracts.

Geographical regions significantly influence the composition of herbal drugs. Dinchev et al. [3] detected prototribestin only in the samples collected from Bulgaria, Turkey, Greece, Macedonia, Iran, and Serbia, but no protodioscin was detected in the samples collected from Vietnam and India. It appeared that this compound could be a marker for the European variety of TT [3]. Lazarova et al. [4] demonstrated that there were considerable differences between the samples collected from the same country; dioscin was not detected in some samples collected from Bulgaria, and the concentrations of the compounds also varied widely. The obtained result could be correlated with the methods used for extraction because furostanol bidesmosides were transformed into their spirostanol monodesmosides analogs during extraction. Lazarova et al. [4] performed the extraction by sonication for 15 min, using 50% aqueous acetonitrile as a solvent, but as shown by Sarvin et al. [18], a longer extraction time (60 min) gave a better yield. The β-Carboline indole alkaloids, i.e., harman, harmine, and harmalol were isolated from fruits, leaves, stems, and roots, but harmaline was only isolated from the roots, stem, and leaves [44]. As can be seen in Table 2, the concentration of protodioscin, prototribestin, dioscin, tribestin, and tribulosin varies within very wide limits depending on the origin of the plant. Differences are noticed between the different organs of the plant. These significant variations in TT composition explain the opposite pharmacological effects obtained in the performed studies. In Figure 2 are presented the chemical structures of the main compounds found in TT, other than the steroidal compounds.

## 3. Pharmacological Properties

### 3.1. Pharmacokinetic Properties of TT Main Compounds

Protodioscin is the dominant component in TT fruits and is considered to be the main pharmacologically active steroidal saponin [3]. Studies regarding the pharmacokinetic characteristics of protodioscin have contradictory results. For example, a recent study published by Zhang et al. [52] concluded that protodioscin had low bioavailability in vivo. However, the same group of authors has shown that after the administration of an extract from *Dioscorea*, the pharmacokinetic profile of protodioscin revealed good bioavailability [53]. Despite multiple in vivo studies with TT, very little is known about the pharmacokinetics of the therapeutically active compounds. There are, however, pharmacokinetic studies for protodioscin and dioscin after the administration of different *Dioscorea* sp. extracts [53,54]. Saponins, due to their amphiphilic molecule, have membrane permeabilizing properties, thus, they could increase the absorption of other compounds. This property is of great importance because toxic effects could appear in patients with multiple conditions who undergo chronic treatments.

### 3.2. Antioxidant Activity

Production of reactive oxygen species (ROS) in the body and their correlation with the incidence of chronic diseases has been largely described in the scientific literature and is already a fact. 

TT extracts contain flavonoids and polyphenol carboxylic acids. The antioxidant activity of these compounds has been convincingly confirmed, based on their ability to donate hydrogen. Polyphenols are capable of scavenging hydroxyl (HO^•^), peroxyl (RO_2_^•^), and superoxide (O_2_^•−^) radicals [55].

**O_2_** → **O^•^_2_^−^** → **H_2_O_2_** → **HO^•^** + **HO^−^** → **2H_2_O** (1)

Nevertheless, the effect of flavonoids varies and is strictly linked to their chemical characteristics and functional groups. Lower scavenging effects were noticed on singlet oxygen, and only for flavonones and phenolic acids [56]. In vitro determinations have proven that TT extracts have antioxidant activity determined using DPPH, ABTS, and FRAP methods. The reported concentration of polyphenols ranges from 0.6% to 3% and the content in flavonoids ranges from 0.04% to 0.5% [57,58]. It has been shown that when fractionated extracts were tested for their antioxidant activity, the ethyl acetate fraction had the strongest DPPH free radical scavenging activity, and the responsible compounds from this fraction were 4,5-di-p-*cis*-coumaroylquinic acid, and 4,5-di-p-*trans*-coumaroylquinic acid [27].

Dutt-Roy et al. [59] observed in an in vivo study that the treatment with TT extracts (part of the plant not specified, ethanol extraction, origin India) increased the activities of catalase and superoxide dismutase, and decreased the malondialdehyde (MDA) concentration. These effects were noticed in diabetic rats and in rats with depression induced by para-chlorophenylalanine (a selective and irreversible inhibitor of tryptophan hydroxylase) [59]. Catalase breaks down hydrogen peroxide (H_2_O_2_) to water and oxygen, and it has an essential role in the protection of cells from ROS. Superoxide dismutase catalyzes the transformation of superoxide anion free radical (O_2_^−^) into oxygen (O_2_) and H_2_O_2_ [60]. MDA is a marker of oxidative stress and is one of the final products of polyunsaturated fatty acids (PUFAs) peroxidation [61].

Studies have shown that STZ-induced diabetes increased oxidative stress, and apparently, TT extracts (plant origin UAE, 70% ethanolic extract) were capable of modulating oxidative stress markers (MDA and GSH) [62].

### 3.3. Sexual Disorders

On the basis of the widespread societal presumption that natural compounds are active in erectile dysfunction, but lack the side effects specific to compounds obtained by chemical synthesis (e.g., phosphodiesterase-5 inhibitors such as sildenafil, tadalafil, etc.), they are often preferred and used for extended periods. Various products containing TT extracts are widely utilized for this purpose, mainly due to the advertising of supplements for professional athletes, based on the alleged effect of testosterone boosting. Existing data in the literature, resulting from in vitro experiments, by analyzing animal models (preclinical studies) and evaluating endpoints from clinical trials on subjects with erectile dysfunction are presented below.

#### 3.3.1. In Vitro Experiments

The main objective of in vitro studies was to evaluate the quality of semen (morphology and viability). In vitro incubation of human spermatozoa with TT extract (origin Iran, part of the plant used not specified, extraction with water) had a beneficial effect on motility and viability. These findings suggest that TT extracts could be further used in the preparation of spermatozoa before in vitro fertilization [63]. An organ bath study of the corpus cavernosum (CC) from rabbits showed that TT extract (origin Korea) produced a concentration-dependent relaxation response. The authors suggested that because the location of action was in the endothelium, the relaxation effect appeared via the NOS pathway [64].

#### 3.3.2. Preclinical Experimental Studies (Animal Models)

Preclinical studies have focused on animal models of human diseases that affect spermatogenesis and androgen secretion (cytotoxic medication that affects the gonads, castration, and diabetes); the effect of TT extracts on spermatogenesis and gonadal steroidogenesis in healthy male subjects, whether or not subjected to standardized physical exertion has also been evaluated.

In adult male Swiss albino mice with reproductive damage induced by cyclophosphamide, TT showed an improvement of epididymal sperm characteristics (motility) and an increase in testosterone levels as compared with the control group [65]. Extracts with TT administered to trained rats (fruit extract, China >70% saponins), diabetic rats (seed extract, Iran), healthy male rats (flowers, Iran) led to a significant increase in testosterone levels as compared with the control [66,67,68]. In healthy Wistar rats, a 70-day supplementation with TT extract influenced spermatogenesis, as shown by the changes in the tubular compartment of the testes (increase in the total tube length, tubular volume, and height of the seminiferous epithelium) [69]. In healthy male rats, a significantly increased testosterone level was confirmed as compared with the control group and positive effects on sexual parameters [68]. The TT extracts (origin Bulgaria) improved sexual behavior in castrated rats (mount frequency, intromission frequency, mount latency, intromission latency, ejaculation latency, and post-ejaculatory interval) [70]. 

#### 3.3.3. Clinical Trials

The analysis of available clinical trials on the effectiveness of TT extracts in men highlights two categories of primary endpoints as follows: Some studies set as their main goal the evaluation of efficacy in erectile dysfunction (erection quality and libido intensity) and others evaluated the change in the basal secretion of testosterone at the end of the study with the initial values of the subjects serving as the control. However, the available studies did not shed light on the controversy regarding the real efficacy of TT. On the one hand, due to the divergent results (when the quantified parameter could be determined accurately such as testosterone and dihydrotestosterone, pituitary gonadotropin levels, etc.); on the other hand, due to the subjective evaluation (especially if the endpoints were based on the self-evaluation of the subjects’ standardized questionnaires such as the International Index of Erectile Function (IIEF), Questionnaire and Global Efficacy Question (GEQ).

Recently, Kamenov et al. [71] evaluated the efficacy and safety of a standardized extract (Tribestan^®^, Sopharma AD-coated tablets containing 250 mg of dry extract equivalent to furostanol saponins not less than 112.5 mg) for the treatment of men with mild to moderate erectile dysfunction and with or without hypoactive sexual desire disorder in a prospective, phase IV, randomized, double-blind, placebo controlled clinical trial in parallel groups. The characteristics of the study can be summarized as follows: dose of three coated tablets per day; sample size of 90 subjects in each group (treated vs. placebo); duration of 12 weeks; primary endpoint, the change in IIEF score at the end of the treatment. The authors showed a significant improvement in erection, libido, and orgasmic function in the treated group, in the absence of any difference in the profile of side effects as compared with the placebo [71].

Santos et al. [72] conducted a prospective, randomized, double-blind study on patients with erectile dysfunction. The treated group received 400 mg of TT extract. There were no significant differences noticed between the placebo and the treated group. The origin of the plant or the method of extraction were not specified.

In contrast to these data, two studies confirmed the beneficial effects after treatment with pharmaceutical products containing TT and other components. The first study showed that after 20 days of supplementation with the dietary supplement “*Tribulus*”, anaerobic muscle power and serum testosterone increased significantly in young men [73]. The other double-blind placebo controlled study in older men with a history of erectile dysfunction and lower levels of total and free testosterone showed high efficacy of a preparation containing TT. The product, called “*Tradamixin*”, consisted of TT, *Alga Eckonia*, D-glucosamine, and N-acetyl-glucosamine, was given daily for two months, and improved libido in elderly men and increased testosterone. It should be noted, however, that in both experiments, there was no certainty that a particular component would have caused those biological benefits or if TT contributed to those effects [74].

In a study conducted on male boxers, the administration of a TT supplement (with >40% saponins) produced no effect on plasma testosterone and dihydrotestosterone. Although the results were inconclusive, the authors suggested that a possible mechanism of action for TT compounds could be related to insulin-like growth factor (IGF-1) and insulin-like growth factor binding protein 3 (IGFBP-3). IGF-1 is a growth hormone that showed the capacity of elevating skeletal muscles and preventing age-related loss of muscle mass [35]. 

Additionally, IGF improves insulin signaling, which could explain the beneficial effects obtained with TT extracts in diabetes, but the exact mechanism of action is not fully known [75].

**Figure 3:**
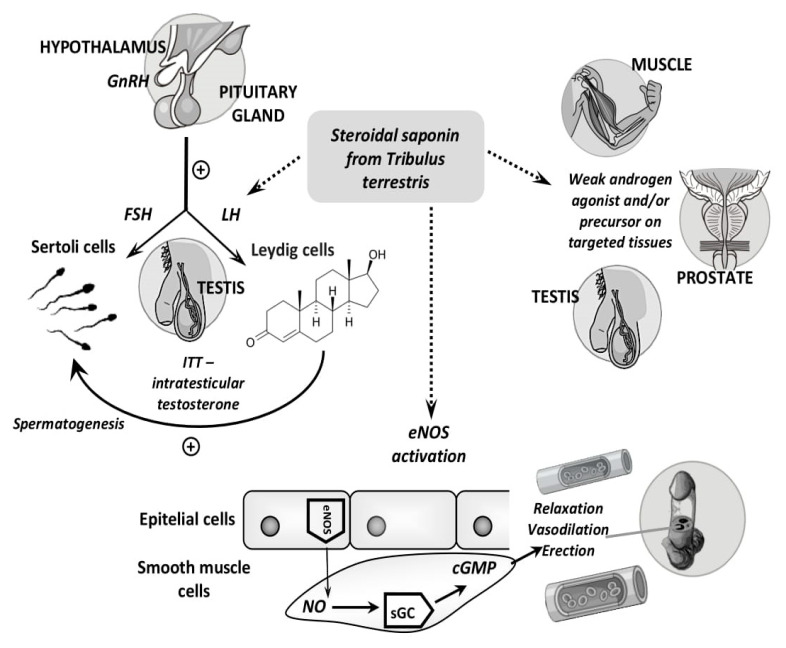
The presumed mechanisms of action responsible for the effects of TT extracts in sexual disorders. GnRH, gonadotropin-releasing hormone; FSH, follicle-stimulating hormone; LH, luteinizing hormone; ITT, intratesticular testosterone; eNOS, endothelial nitric oxide synthase; NO, nitric oxide; sGC, soluble guanylate cyclase; and cGMP, cyclic guanosine monophosphate.

The booster effects of TT extracts have been confirmed by some authors, both in experimental research and in clinical studies, as shown above, but are questioned by others. The available data on the mechanisms underlying the use in sexual disorders can be summarized as follows (Figure 3): steroidal saponins from TT increase the endogenous testosterone levels, due to an indirect action, i.e., the LH-type action of the steroidal saponosides or a weak androgenic agonist type action [13], but these mechanisms are denied by others [76,77]. Luteinizing hormone (LH) regulates the expression of 17β-hydroxysteroid dehydrogenase, which is the enzyme that transforms androstenedione into testosterone [78]. In addition, the antioxidant effect could contribute to the booster action of TT, knowing that oxidative stress is linked to endothelial dysfunction. Nitric oxide mediates the formation of cyclic guanosine monophosphate (cGMP); this mechanism could promote erection by vasodilation and increased blood supply to the corpora cavernosa [64,79]. In oxidative stress, the reactive oxygen species and advanced end glycation products react with nitric oxide in the vasculature forming reactive nitrogen species, contributing to the pathogenesis of erectile dysfunction [80]. Furthermore, different studies have shown that TT extracts are efficient in women with sexual disorders by having a favorable action in clinical trials on hypoactive sexual desire in women, as well as in the control of menopausal transition symptoms [81,82,83]. 

Given the testosterone boosting action of the extract, research has been performed to evaluate if the consumption of TT extracts could influence the doping tests of athletes regarding the urinary testosterone/epitestosterone TS/ET ratio limit of 4:1 (World Anti-Doping Agency) [84].

The in vitro and in vivo studies are briefly presented in Table 3, where pharmacological actions related to sexual disorders have been evaluated.

### 3.4. Antibacterial Activity

There are several in vitro studies that have revealed the antibacterial potency of TT total or fractionated extracts on Gram-negative and Gram-positive bacterial strains. Among the Gram-positive bacteria, facultative anaerobe strains such as *Staphylococcus aureus, Streptoccocus mutans, Streptococcus sanguinis, Actinomyces viscosus, Enteroccocus faecalis,* and *Bacillus subtilis* were susceptible and among the Gram-negative bacteria *Escherichia coli, Salmonella typhi, Proteus mirabilis,* and *Klebsiella pneumoniae* were susceptible [87,88,89,90,91,92]. It is still unclear which components are responsible for the antibacterial activity, but alkaloids contribute to the general antibacterial effect of the total extracts [88]. The antibacterial effects of saponins are well documented and the mechanism of action is based on the destruction of the cell membrane, leading to cell death (bactericidal effect), probably due to their amphiphilic nature and their surfactant properties. In addition, it was noticed that saponins could modulate ion channels, influencing the membrane potential [93,94]. Kianbakht and Jahaniani [92] found that the antibacterial activity of extract from TT roots was lower than the activity of the extracts obtained from the fruits and stems plus leaves. Although the authors did not provide a phytochemical profile of the extracts, we have shown in Table 2 that furostanol and spirostanol saponins were mainly identified and quantified in the aerial parts of TT rather than in the roots. However, alkaloids were identified in all organs. These results suggest that the antibacterial activity of TT is correlated mostly with the saponin content. Flavonoid fractions from TT leaves and fruits have also been proven to have antibacterial activity against *E. coli*, *Salmonella*, *Staphylococcus aureus*, and *Streptococcus* [39,40].

A recently published paper demonstrated the quorum quenching activity of TT (origin India) root extracts on *Chromobacterium violaceum*, *Serratia marcescens,* and *Pseudomonas aeruginosa* strains. The main compound was found to be ß-1, 5-*O*-dibenzoyl ribofuranose [51].

### 3.5. Antihyperglycemic Effect

#### 3.5.1. In Vitro Determinations

Studies conducted with extracts from TT have been shown to inhibit the activity of alpha-glucosidase and alpha-amylase in vitro. Alpha-glucosidase and alpha-amylase are enzymes involved in the hydrolysis of carbohydrates. Alpha-amylase breaks down the oligosaccharides into disaccharides and alpha-glucosidase breaks down the disaccharides into absorbable monosaccharides. Inhibition of the activity of these enzymes has been proven to reduce postprandial hyperglycemia in diabetic patients. The TT extracts exhibited a relatively higher inhibition capacity on alpha-amylase than on that of alpha-glucosidase [95]. The activity of the total extract was higher than the activity of isolated saponin, meaning that there are other constituents in the TT extract that act synergistically. As reported by Song et al., cinnamic acid amides also have the capacity to inhibit the activity of alpha-glucosidase [36]. Ponnusamy et al. [96] concluded that TT had a lower capacity of inhibition of the activity of alpha-glucosidase as compared with other extracts.

#### 3.5.2. Preclinical Studies

**Figure 4:**
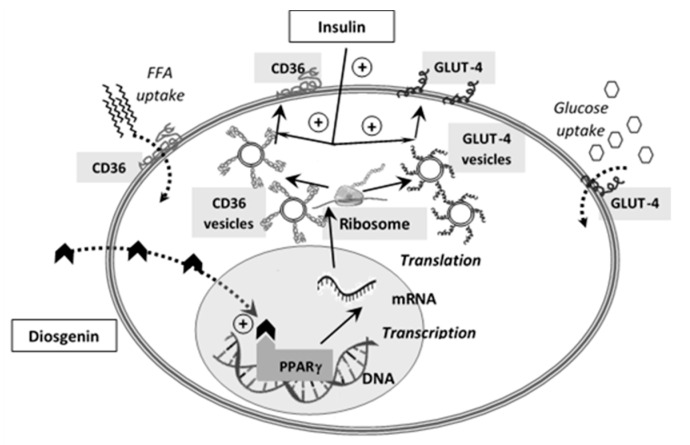
The presumed mechanism of diosgenin stimulation of PPARγ receptors. PPARγ, peroxisome proliferator-activated receptor gamma; DNA, deoxyribonucleic acid; mRNA, messenger ribonucleic acid; FFA, free fatty acids; GLUT-4, glucose transporter 4; and CD36, cluster of differentiation 36 (fatty acid translocase).

In vivo animal studies are in concordance with the in vitro studies, as it was shown that the saponins from TT administered to rats were able to delay the postprandial hyperglycemia by inhibiting alpha-glucosidase [97]. Studies on diabetic rats and glucose-loaded rabbits have shown that TT extracts are also capable of reducing fasting blood glucose levels, which suggests that the active compounds have multiple mechanisms of action [98,99,100]. Although the majority of the preclinical research for TT extracts was conducted on diabetic rats in order to evaluate the effect on different complications caused by diabetes, mostly related to sexual disorders, all studies reported the antihyperglycemic effect of TT extracts [67,85,86]. Diosgenin was shown to promote insulin secretion and influence beta cell regeneration in STZ-induced diabetes in rats through PPARγ activation in adipose tissue and oxidative stress modulation [101,102]. Stimulation of PPARγ nuclear receptors as a likely mechanism of the antihyperglycemic effect of diosgenin could explain the insulin-sensitizing action by altering the free fatty acid/glucose ratio by facilitating their intracellular uptake into muscle and adipose tissue. Intracellular uptake of both glucose and free fatty acids could be the consequence of stimulating the expression of GLUT-4 (glucose transporter 4) and CD36 (cluster of differentiation 36 or fatty acid translocase) as a result of PPARγ receptor activation (Figure 4).

Alkaloids could act synergistically with the steroidal saponins, as it was shown that imidazolidine derivatives stimulate insulin secretion by activation of imidazoline receptor type 3 binding sites in the pancreatic beta cells [103].

#### 3.5.3. Clinical Studies

Samani et al. [104] conducted a double-blind, randomized placebo controlled clinical trial that included ninety-eight women. The study concluded that TT extract significantly lowered the blood glucose level of diabetic patients as compared with the placebo group. Another study conducted by Ramteke et al. [105] included 100 patients with diabetes mellitus and microalbuminuria. The results showed that the group treated with an ayurvedic preparation that contained TT had significantly lower blood glucose after the treatment as compared with the initial blood glucose level and the microalbuminuria was also reduced.

Recent research suggests that there is a correlation between testosterone levels and type 2 diabetes and that low testosterone levels in men predict a high risk of type 2 diabetes [106]. The direct and indirect androgenic action of TT extracts could also contribute to the improvement in the glycemic profile of diabetic patients, as it is known that androgens increase carbohydrate tolerance and promote glycogenesis [107]. 

Table 4 summarizes the most relevant results obtained in pharmacological studies related to the antihyperglycemic effect of TT.

### 3.6. Anti-Inflammatory Properties

#### 3.6.1. In Vitro Studies

Several studies have demonstrated that extracts of TT have anti-inflammatory activities. The primary mechanisms involved are thought to be downregulation of inflammatory pathway protein NFκB [46]. The extract used was standardized in tribulusterine (aqueous extract with 0.54 mg% tribulusterine, origin India, part of the plant used not specified). Because the protein NFκB is also a mediator of cell cycle and cell survival, it has been shown that TT extracts can induce apoptosis in human liver cancer cells by inhibiting the NFκB signaling pathway (aqueous extract from fruits, origin Korea) [108]. Research has also shown that the extracts have an anti-inflammatory effect even in the topical application by affecting modulation of the calcium channels Orai-1 and TRPV3, as well as by inhibiting mast cell activation (ethanolic extract from fruits, origin Korea) [43]. The only compound identified in TT extract was rutin. Lee et al. [48] assessed the anti-inflammatory effects of tribulusamide D isolated from the fruits of TT in an in vitro study (origin Korea). They suggested that the effect occurred through the downregulation of enzymes responsible for the production of cytokines and inflammatory mediators. Hong et al. [109], demonstrated that TT fruits extract (origin Korea) inhibited the COX-2 activity. Other in vitro studies have shown that TT extracts have anti-inflammatory effects [39,110].

#### 3.6.2. In Vivo Studies

Animal experiments have confirmed the anti-inflammatory effects demonstrated in vitro. Mohammed et al. [111] showed that the methanolic extract from the aerial parts of TT (origin Sudan) and the chloroformic fraction had significant anti-inflammatory effects in rat paw edema induced with carrageenan as compared with the untreated group. The anti-inflammatory effect of a flavonoid fraction from TT leaves was also evaluated in an ear swelling model induced by xylene in mice. The study demonstrated that the flavonoid fraction reduced the swelling degree in a dose-dependent manner [39]. Qiu et al. [112] tested terrestrosin D on bleomycin-induced inflammation in mice. They concluded that TT administration suppressed the inflammatory and fibrotic changes induced by bleomycin in the lungs.

### 3.7. Action on the Central Nervous System

The β-Carboline indole alkaloids are known to be monoamine oxidase inhibitors (MAOIs), primarily MAO-A, as they prevent biogenic amine from binding to the active site of the MAO molecule and undergoing deamination. Consequently, their presence in TT is thought to have been responsible for the unusual locomotory disturbance in sheep that grazed in areas with TT [45,113]. If this action is maintained in humans, special precautions should be taken in patients under treatment with monoamine oxidase inhibitors.

In several studies, the neuroprotective effect has been demonstrated and several mechanisms of action have been proposed. Chaudary et al. [114] demonstrated the neuroprotective effect of TT extracts (fruits part of the plant, origin Pakistan) in aluminum chloride-induced Alzheimer’s disease in rats. Biochemical and behavioral parameters improvement were connected with the antioxidant activity of the extract and also with the chelating properties of flavonoids. Song et al. [115] evaluated the anticonvulsant effect of protodioscin on a pilocarpine-induced convulsion model in mice and suggested that the effect was modulated through the GABAergic system.

Part of the previously mentioned effects of TT extracts is mediated through the central nervous system, and therefore are not included in the present section. These include the modulation of pituitary gonadotropin secretion. The toxic effects observed in sheep also involve the modulation of the GABAergic and dopaminergic system and are further presented.

### 3.8. Toxicological Studies

#### 3.8.1. In Vitro Studies

Evaluation of toxicological effects in vitro has demonstrated that TT extracts (part of the plant not specified, origin Turkey) have estrogenic and genotoxic effects [116].

#### 3.8.2. Preclinical Experimental Studies (Animal Models)

Hepatogenous photosensitivity appeared after 11 days in sheep fed with a mixture of TT and alfalfa (*Medicago sativa*). The symptoms included depression, jaundice, weight loss, conjunctivitis, and also the reddening of the muzzle, nose, ears, and eyelids [117]. The study conducted by Gandhi et al. [118], on diabetic rats, was inconclusive with respect to the nephrotoxic effects of TT extract (50 mg hydroalcoholic extract/kg with 45% saponins). Although an improvement in kidney function was expected after the treatment, no improvement was noticed [118]. Bourke [119] reported that a specific, irreversible, asymmetrical locomotor disorder appeared in sheep that ingested large quantities of TT. Administration of levodopa to the affected and nonaffected sheep, followed by the removal of the striatum and the quantification of dopamine and 3,4-dihydroxyphenylacetic acid, led the author to the conclusion that chronic intake of large quantities of TT caused a malfunction of the striatal presynaptic receptor, affecting the nigrostriatal pathway. The same author, along with other scientists, continued the research in this field and indicated harmane and norharmane as two possible neurotoxins [45].

Acute and subacute toxicity tests were performed by Hemalatha and Hari [120] with butanolic extract from TT fruits (origin India). No signs of significant toxicity were noticed. Also, El-Shaibany et al. [100] concluded that there were no toxic symptoms, deaths or behavioral changes in an acute toxicity study in rabbits treated with TT aerial parts extract (origin Yemen).

#### 3.8.3. Case Reports

Talasaz et al. [121] reported a severe case of nephrotoxicity in a 28-year-old man, after the consumption of TT water. There is also a published case presentation in which a 36-year-old man, who consumed a herbal supplement based on a TT extract, was diagnosed with a 72-hour priapism [122]. It was presumed that the priapism was caused by TT supplement, and no further analysis of the supplement was performed; therefore, a pertinent conclusion cannot be drawn regarding this side effect, i.e., if it was caused by the extract found in the supplement or by an unknown compound with which the supplement was impurified. Another reported case of toxicity caused by consumption of TT supplements was that of a 30-year-old male, diagnosed with hyperbilirubinemia, cholestasis, and bilirubin-induced toxic acute tubular necrosis [123]. As in the previous case, the analysis of the supplement was not performed. 

The toxicity of TT extracts has not been fully evaluated, and the toxic compounds have not been properly identified. 

With respect to the reported cases of toxicity, no clinical trial in which TT-based products were administered, have reported these side effects. Particular attention should be given to the herbal supplements, and an elaborate analysis should be performed in order to identify the toxic compounds. There is a constant risk of adulteration of food supplements, primarily when these are used for their anabolic effects. There is also the possibility of trace metal accumulation in herbal drugs. A single research article was found that analyzed the content of some essential and trace elements in TT organs [124]. Although the results did not indicate toxic concentrations in the samples, a routine analysis of these elements should be performed for the food supplements. Antinutritional factors (hydrocyanic acid, phytate, nitrate, and oxalate) in TT leaves were also identified [125]. Seven compounds (listed in Table 5) from TT have a toxicological profile in the U.S. National Library of Medicine [126]. Harmine was the only compound found to have a complete toxicological profile. Effects of toxic doses are tremor, sleepiness, nausea or vomiting (man), excitement, mydriasis, dyspnea and ataxia (rabbit), and excitements (mouse).

Considering all of the above information, a complete analysis of the supplements should be performed when a toxicity case is reported.

## 4. Conclusions

Different phytochemical profiles of the herbal drugs from TT, highlighted both in the concentration of the main active compounds and in the absence of some active compounds, explain the major differences in the therapeutic effects reported over the years in the literature. The main pharmacological research on TT has been focused on sexual disorders, but other important effects have been demonstrated in vitro and in vivo studies, i.e., anti-hyperglycaemic, anti-inflammatory, antioxidant, and antibacterial. Toxicological studies, although limited, have highlighted the risk of nephrotoxicity following the administration of TT supplements. However, additional studies are needed to determine some of the still unknown molecular mechanisms of action of the therapeutic active compounds found in *Tribulus* extracts. Although TT has been extensively researched, further studies are needed in order to clarify important aspects such as a more accurate correlation between the phytochemical and pharmacological profiles, pharmacokinetic studies of the most important compounds, as well as the evaluation of possible pharmacokinetic and pharmacodynamic interactions with other compounds. Researchers should provide full information on the plant origin and the tested organ. Methods for standardization are necessary in order to achieve reproducible results. To date, it seems that the chemical compounds found in TT are capable of activating multiple pathways, hence, the various effects. 

## Figures and Tables

**Table 1:** Previous reviews.

Year of the Review	Main Topic	Years Surveyed	Limitations	Reference
2005	Phytochemistry and pharmacology	<2004		[6]
2014	TT supplements	NS		[1]
2014	Phytochemistry and pharmacology		short review	[7]
2014	Phytochemistry and pharmacology	NS	short review	[8]
2016	Analysis of human and animal evidence	1968–2015		[2]
2016	Phytochemistry	NS	Only the composition of fruits was discussed	[9]
2016	Phytochemistry and pharmacology	NS		[10]
2017	Phytochemistry and pharmacology	NS		[11]
2018	Male infertility		short review	[5]
2019	Phytochemistry and ethnomedicine	NS	brief presentation of constituents	[12]
2019	Male infertility	NS		[13]
2019	Phytochemistry and pharmacology	1965–2017		[14]
2020	Phytochemistry and pharmacology	NS	the review is based mostly on Ayurvedic preparation The pharmacological effects are briefly presented	[15]

NS, not specified.

**Table 2:** Chemical compounds identified in *Tribulus terrestris* (TT).

Compound	Chemical Formula	Plant Part	Conc. mg/100 g	Plant Origin	References
**Furostanol Saponins**					
Protodioscin	C_51_H_84_O_22_	aerial parts	109–1530	Bulgaria	[3,4,16,17]
		leaves	1000–1330		
		stem	19–27		
		fruits	240–500		
		aerial parts	340–1000	Turkey	[3]
		fruits	10–60		
		aerial parts	220–790	Greece	[3]
		aerial parts	420–990	Macedonia	[3]
		aerial parts	200	Serbia	[3]
		aerial parts	560	Georgia	[3]
		aerial parts	3	Vietnam	[3]
		fruits	1		
		fruits	63–89	China	[17]
		stem	24	India	[17]
		aerial parts	190	Russia	[18]
Neoprotodioscin	C_51_H_86_O_22_	aerial parts	NS	Bulgaria	[16]
Prototribestin	C_45_H_73_NaO_20_S	aerial parts	130–2200	Bulgaria	[3,4,16,19]
		fruits	21–28		
		leaves	700		
		stems	40		
		aerial parts,	310–1000	Turkey	[3]
		fruits	17–65		
		aerial parts	220–790	Greece	[3]
		aerial parts	420–990	Macedonia	[3]
					[3]
		aerial parts	170	Serbia	[3]
		aerial parts	240	Georgia	[3]
Neoprototribestin	C_45_H_75_NaO_20_S	aerial parts	NS	Bulgaria	[16]
Terestrinin A	C_33_H_48_O_9_	fruits	NS	China	[20]
Terestrinin B	C_60_H_95_O_30_	root	NS	Georgia	[21]
		fruits	NS	China	[20]
Terrestrinin D	C_33_H_50_O_10_	fruits	5.6	China	[22,23]
Terestrinin J-T		whole plant	NS	China	[24]
Terestroside A		root	NS	Georgia	[21]
Terrestrosin K	C_51_H_82_O_24_	fruits	1.27	China	[22]
Terrestrosin I	C_51_H_84_O_25_	whole plant	NS	China	[23,24]
		fruits			
Tribufuroside D	C_45_H_74_O_21_	fruits	NS	China	[23,25]
Tribufuroside E	C_45_H_74_O_21_	fruits	NS	China	[23,25]
Tribulosaponin A	C_51_H_84_O_21_	fruits	NS	China	[26]
Polianthoside D	C_56_H_92_O_29_	root	NS	Georgia	[21]
		fruits	59.6	China	[22]
**Spirostanol Saponins**					
Dioscin	C_45_H_72_O_16_	aerial parts	NS	Egypt	[27]
		aerial parts	60	Russia	[18]
		fruits, leaves, stem	10–43	Bulgaria	[3,4,28]
		aerial parts	6–13	Turkey	[3]
		fruits	1–2		
		aerial parts	26–31	Greece	[3]
		aerial parts	13–15	Macedonia	[3]
		aerial parts	87	Serbia	[3]
		aerial parts	8	Georgia	[3]
Tribestin	C_39_H_61_NaO_14_S	aerial parts	2–220	Bulgaria	[3,28]
		fruits	0.9–3.4		
		leaves	62		
		aerial parts	6.8–28	Turkey	[3]
		fruits	0.5–1		
		aerial parts	24	Greece	[3]
		aerial parts	7.3–10	Macedonia	[3]
		aerial parts	210	Serbia	[3]
		aerial parts	6	Georgia	[3]
Diosgenin	C_27_H_42_O_3_	NS	NS	China	[29]
		NS	NS	Ukraine	[30]
		fruits	86	India	[31]
Tribulosin	C_55_H_90_O_25_	aerial parts	0.1–7.7	Bulgaria	[3]
		fruits	2.6		
		leaves	0.8		
		stem	1.7		
		aerial parts	0.03–1.7	Turkey	[3]
		fruits	0.14		
		aerial parts	1.3–2.4	Greece	[3]
		aerial parts	0.68	Macedonia	[3]
		aerial parts	2.24	Serbia	[3]
		aerial parts	0.56	Georgia	[3]
		aerial parts	22	Vietnam	[3]
		fruits	420		
		fruits	1	India	[3]
		leaves	644		
		stem	185		
		whole plant	NS	India	[32]
Tigogenin	C_27_H_44_O_3_	fruits	0.05	China	[22,29,33]
Terestrinin U		whole plant	NS	China	[24]
Gitogenin	C_27_H_44_O_4_	NS	NS	China	[33]
Hecogenin	C_27_H_42_O_4_	fruits	NS	Taiwan	[34]
		fruits	0.4	China	[22]
Agovoside A		fruits	NS	China	[20]
Prosapogenin B		aerial parts	NS	Egypt	[27]
25R-5a-Spirost-3,6,12-trione	C_27_H_39_O_5_	NS	NS	China	[33]
25R-Spirost-4-ene-3,12-dione	C_27_H_40_O_4_	NS	NS	China	[33,35]
25R-Spirost-4-ene-3,6,12-trione	C_27_H_38_O_6_	NS	NS	China	[33,35]
**Cinnamic Acid Amides**					
Coumaroyltyramine	C_17_H_17_NO_3_	fruits	NS	Taiwan	[34,36,37]
		fruits	NS	China	
Ferulic acid		fruits	NS	Taiwan	[34]
Feruloyloctopamine	C_18_H_19_NO_5_	fruits	NS	China	[36]
**Quinic Acid Derivatives**					
5-p-cis-coumaroylquinic acid	C_16_H_18_O_8_	aerial parts	NS	Egypt	[27]
5-p-*trans*-coumaroylquinic acid		aerial parts	NS	Egypt	[27]
4,5-Di-p-*trans*-coumaroylquinic acid		aerial parts	NS	Egypt	[27]
4,5-Di-p-*cis*-coumaroylquinic acid		aerial parts	NS	Egypt	[27]
**Flavonoids**					
Tribuloside	C_30_H_26_O_13_	leaves, fruits	NS	India	[38]
Kaempferol	C_15_H_10_O_6_	leaves, fruits	18	India	[31,38]
Astragalin (kaempferol 3-glucoside)	C_21_H_20_O_11_	leaves, fruits	NS	India	[38]
Kaempferol 3-rutinoside	C_27_H_30_O_15_	leaves, fruits	NS	India	[38]
Kaempferol-3- gentiobioside	C_27_H_30_O_16_	fruits leaves	NS	China	[39,40]
Rutin	C_27_H_30_O_16_	leaves	NS	Mauritania	[4,41,42,43]
		fruits, leaves	NS	India	
		fruits, leaves	70–250	Bulgaria	
		fruits	NS	Korea	
		NS	NS	Ukraine	[30]
Quercetin	C_15_H_10_O_7_	fruits, leaves	NS	India	[42]
Quercetin-3-*O*-arabinosyl galactoside Isorhamnetin-3-glucoside	C_26_H_28_O_16_	fruits leaves	NS	China	[39,40]
Quercetin-3-*O*-sophoroside-7-*O*-glucoside	C_33_H_40_O_21_	leaves	NS	China	[39]
Quercetin-3- gentiobioside	C_27_H_30_O_17_	fruits, leaves	NS	China	[39,40]
Quercetin 3,7-diglucoside	C_27_H_30_O_17_	fruits, leaves	NS	China	[39,40]
Isoquercitrin	C_21_H_20_O_12_	fruits, leaves	NS	China	[39,40]
Luteolin-7-*O*-β-D- glucoside	C_30_H_18_O_11_	leaves	NS	China	[39]
Isorhamnetin-3-glucoside	C_22_H_22_O_12_	leaves	NS	China	[39]
Apiotribosides A-D		roots	NS	Georgia	[21]
**Alkaloids**					
Harmine	C_13_H_12_N_2_O	fruits	14	India	[31]
		fruits, stem, leaves, roots	NS	Turkey	[44]
Harmane	C_12_H_10_N_2_	fruits, stem, leaves, roots	NS	Turkey	[44]
		aerial parts	NS	Australia	[45]
Harmalol	C_12_H_12_N_2_O	fruits, stem, leaves, roots	NS	Turkey	[44]
Harmaline	C_13_H_14_N_2_O	stem, leaves, roots	NS	Turkey	[44]
Norharmane	C_11_H_8_N_2_	aerial parts	NS	Australia	[45]
Tribulusterine	C_16_H_12_N_2_O_2_	fruits	NS	Taiwan	[34]
		not specified	NS	India	[46]
n-Caffeoyltyramine		fruits	NS	Korea	[36,47]
		fruits		China	
Perlolyrine	C_16_H_12_N_2_O_2_	not specified	NS	India	[46]
**Amides and Lignanamides**					
Terrestribisamide	C_13_ H_18_NO_5_	fruits	NS	Taiwan	[34]
Tribulusamide A	C_36_H_36_N_2_O_8_	fruits	NS	China	[37]
Tribulusamide B	C_36_H_34_N_2_O_9_	fruits	NS	China	[37]
Tribulusamide D	C_17_H_15_NO_5_	fruits	NS	Korea	[48]
Tribulusamide C	C_18_H_15_NO_6_	fruits	NS	China	[49]
**Fatty Acids and Fatty Acid Esters**					
Oleic acid	C_18_H_34_O_2_	stem	NS	Pakistan	[50]
Palmitic acid	C_16_H_32_O_2_	stem	NS	Pakistan	[50]
6,9,12,15-Docosatetraenoic acid, methyl ester	C_23_H_38_O_2_	stem	NS	Pakistan	[50]
Pentadecanoic acid, 14-methyl-, methyl ester	C_17_H_34_O_2_	stem	NS	Pakistan	[50]
9,12-Octadecadienoic acid, methyl ester (E,E)-	C_19_H_34_O_2_	stem	NS	Pakistan	[50]
**Phytosterols**					
β-sistosterol-D-glucoside	C_35_H_60_O_6_	whole plant	NS	India	[32]
Stigmasterol	C_29_H_48_O	stem	NS	Pakistan	[50]
**Other Compounds**					
ß-1, 5-*O*-dibenzoyl ribofuranose	C_19_H_18_O_7_	roots	NS	India	[51]
1,3-Benzenedicarboxylic acid, bis(2-ethylhexyl) ester	C_24_H_38_O_4_	stem	NS	Pakistan	[50]
Apiol	C_12_H_14_O_4_	stem	NS	Pakistan	[50]
Octacosane	C_28_H_58_	stem	NS	Pakistan	[50]
Heptacosane	C_27_H_56_	stem	NS	Pakistan	[50]

Concentration is expressed in mg/100 g DW (dry weight). NS, not specified or the concentration could not be calculated using the given data in research paper.

**Table 3:** In vitro and in vivo studies regarding the efficacy of TT extracts in sexual disorders and their design evaluation.

Herbal Drug and Subjects	Assay/Parameters	Outcome of Treated Group	Study Design Evaluation	Reference
In Vitro Studies				
Organ bath study of the *corpus cavernosum* from	Relaxation level	Concentration-dependent relaxation response	Part of the plant: NO	Kam et al. (2012)
male rabbits			Origin: NO	[64]
			Phytochemical analysis: NO	
			Control group: NO	
			Appropriate Statistical analysis: YES	
Human sperm from 40 healthy volunteers	Motility analysis	Motility ↑ * after 60 minutes of incubation	Part of the plant: NO	Khaleghi et al. (2017)
TT extract	Sperm viability analysis	Viability ↑ * in a dose-dependent manner after 120 minutes of incubation	Origin: YES	[63]
	Determination of DNA fragmentation	No effect on DNA fragmentation of human sperm in vitro	Phytochemical analysis: NO	
			Control group: YES	
			Appropriate statistical analysis: YES	
In Vivo Animal Studies				
Male adult Sprague Dawley rats, castrated and normal	Sexual behavior studies: MF, IF, ML, IL, EL, PEI	Treatment of castrated rats (with testosterone or TT extract) showed increase in prostate weight and ICP that were statistically significant	Part of the plant: NCS	Gauthaman et al. (2002)
TT extract	ICP	Mild to moderate improvement of sexual behavior parameters	Origin: YES	[70]
			Phytochemical analysis: NCS	
			Control group: YES	
			Positive control group: YES	
			Appropriate statistical analysis: YES	
Male Sprague Dawley rats	ICP	ICP concentration-dependent increase in TT treated group*	Part of the plant: NCS	Kam et al. (2012)
TT extract, *Cornus officinalis* extract and a mixture of both	cAMP, cGMP in corpus cavernosum	cAMP ↑* in the group treated with the mixture	Origin: YES	[64]
		cGMP no significant difference as compared with the control	Phytochemical analysis: NO	
			Control group: YES	
			Positive control group: NO	
			Appropriate statistical analysis: YES	
-Male rats	Morphometric analysis	Testicular weight ↑*	Origin: YES	Oliveira et al. (2015)
TT fruit extract and fractions	Gonadosomatic index	Gonadosomatic index increased in the group supplemented with ethanolic extract	Part of the plant: YES	[69]
	Sperm quality analysis: motility,	-Nuclear, cytoplasmic, and individual volume of Leydig cells increased in supplementation with hexanic and aqueous fractions	Phytochemical analysis: NO	
	sperm count,	The extract influenced the spermatogenesis	Control group: YES	
	morphology, viability		Positive control group: NO	
			Appropriate statistical analysis: YES	
				
Male Wistar rats with STZ-induced diabetes (55 mg/kg)	Sperm characteristics, morphology	TT restored antioxidant enzyme activity in testis	Part of the plant: YES	Tag et al. (2015)
TT fruit extract	Body and genital organ weight	Improved lipid profile content in serum	Origin: YES	[85]
	Serum testosterone, FSH, LPO level in testicular homogenate	TT treatment decreased testis tubular damage and restored it to normal morphology.	Phytochemical analysis: YES (identification reactions)	
	Activity of testicular SOD		Control group: YES	
	Testicular CAT activity		Positive control group: YES	
	GPx, GST		Appropriate statistical analysis: YES	
Male Wistar rats with STZ-induced diabetes (50 mg/kg)	Testosterone	Sperm motility, sperm count, percentage of sperms with normal morphology ↑*	Part of the plant: YES	Ghanbari et al. (2016)
TT seed extract	Sperm analysis: morphology, count and motility	Testosterone ↑*	Origin: NO	[67]
			Phytochemical analysis: NO	
			Control group: YES	
			Positive control group: NO	
			Appropriate statistical analysis: YES	
Male Sprague Dawley rats	Time to exhaustion of over trained rats	Performance (time to exhaustion) ↑*	Origin: YES	Yin et al. (2016)
TT fruit extract (saponins >70%)	Serum testosterone, corticosterone, AR, IGF-1R in liver, gastrocnemius, and soleus	Increase in body weights, relative weights, and protein levels of gastrocnemius	Part of the plant: YES	[66]
		Testosterone ↑*	Phytochemical analysis: YES (UHPLC-Q-TOF/MS)	
		AR ↑*	Control group: YES	
		IGF-1R ↓#	Appropriate Statistical analysis: YES	
Adult male Swiss albino mice	SOD, CAT, GPx,	SOD, CAT, GST ↓#	Part of the plant: YES	Pavin et al. (2018)
TT fruit extract	GR, GST, GSH, 17β-HSD	GPx ↑#	Origin: YES	[65]
	Plasma testosterone	17β-HSD activity in treated group was not statistically significant different as compared with the control group	Phytochemical analysis: YES (UHPLC-Q-TOF/MS)	
	Semen analysis:	Testosterone ↑	Control group: YES	
	motility, vigor, membrane integrity	Motility ↑#	Positive control group: YES	
	Histology of testes	No significant modifications in testicular architecture	Appropriate statistical analysis: YES	
				
Male Wistar rats	Sperm analysis: sperm count, viability, motility	Testosterone, LH ↑*	Part of the plant: YES	Haghmorad
TT flower extract and	Serum testosterone, LH, FSH levels	All the treatment groups had higher number of Leydig, spermatogonia and spermatid cells	Origin: YES	et al. (2019)
*Anacyclus Pyrethrum* dried root extract	Histological analysis of Leydig and Sertoli cells, spermatogonia, and spermatid cell numbers measure		Phytochemical analysis: NO	[68]
			Control group: YES	
			Positive control group: NO	
			Appropriate statistical analysis: YES	
				
Sprague Dawley rats with type 2 diabetes induced with high-fat and high-sugar feeding and STZ (30 mg/kg)	ICP, MAP	ICP, ICP/MAP ↑ *	Part of the plant: NCS	Zhang et al. (2019)
Gross saponins of TT (GSTT)	eNOS expression level	Nitric oxide ↑*	Origin: YES	[86]
	Nitric oxide level	ROS ↓*	Phytochemical analysis: NCS	
	cAMP expression level	No significant difference between the GSTT group and the sildenafil group in increasing cGMP levels	Control group: YES	
	ROS levels		Positive control group: YES	
			Appropriate statistical analysis: YES	
Clinical Studies				
20–36-Year-old men	Testosterone, androstenedione, LH levels in the serum were measured before and after treatment (24, 72, 240, 408, and 576 h)	No significant difference between TT supplemented groups and the control in the serum testosterone, androstenedione, and LH	Part of the plant: YES	Neychev and Mitev (2005)
TT extract			Origin: YES	[76]
			Phytochemical analysis or standardization: YES	
			Placebo group: YES	
			Randomization: YES	
			Double-blind: NCS	
			Appropriate statistical analysis: YES	
Australian elite male rugby league players	Strength, fat free mass	No significant changes	Part of the plant: NCS	Rogerson et al. (2007)
	Urinary T/E ratio	No changes in urinary T/E ratio	Origin: YES	[77]
			Phytochemical analysis or standardization: YES	
			Placebo group: YES	
			Randomization: YES	
			Double-blind: YES	
			Appropriate statistical analysis: YES	
20–22-Year-old athletes	CK, testosterone	CK ↑*	Part of the plant: NCS	Milasius et al. (2009)
TT capsules	Anaerobic alactic muscular power	Testosterone ↑* during the first half (10 days) of the experiment	Origin: NCS	[73]
	Anaerobic alactic glycolytic power	Anaerobic alactic muscular power ↑*	Phytochemical analysis or standardization: NCS	
		Anaerobic alactic glycolytic power ↑*	Placebo group: YES	
			Randomization: NO	
			Double-blind: NO	
			Appropriate statistical analysis: YES	
Double-blind, randomized trial	IIEF, SQolM,	IIEF ↑*	Part of the plant: NCS	Iacono et al. (2012)
Male patients > sixty years with	Testosterone levels after 60 days of treatment,	SQolM ↑*	Origin: NCS	[74]
reduced libido, with or without erectile dysfunction (ED)	Side effects	TT level increased	Phytochemical analysis or standardization: NCS	
Treatment with “Tradamixina”, tadalafil		No side effects (headache,	Placebo group: NO	
		nasopharyngitis,	Randomization: YES	
		back pain,	Double-blind: YES	
		dizziness,	Appropriate statistical analysis: NO	
		dyspepsia) were observed		
				
Prospective, randomized, double-blind, placebo controlled study	IIEF and serum testosterone were obtained before randomization and after 30 days of study	No effects as compared with the placebo	Part of the plant: NO	Santos et al. (2014)
Healthy men, spontaneously complaining of ED, ≥40 years of age			Origin: NO	[72]
TT extract			Phytochemical analysis or standardization: NO	
			Placebo group: YES Randomization: YES	
			Double-blind: YES	
			Appropriate statistical analysis: YES	
Randomized, double-blind, placebo controlled clinical trial study	FSFI score	FSFI ↑*	Part of the plant: YES	Akhtari et al. (2014)[83]
Women with hypoactive sexual desire disorder			Origin: YES	
TT leaves extract			Phytochemical analysis or standardization: NCS	
			Placebo group: YES	
			Randomization: YES	
			Double-blind: YES	
			Appropriate statistical analysis: YES	
Prospective, randomized, double-blind, placebo controlled clinical trial	IIEF score	IIEF score ↑*	Part of the plant: YES	Kamenov et al. (2017)
Male with mild to moderate ED	GEQ responses	GEQ responses ↑*	Origin: YES	[71]
TT product: Tribestan®,			Phytochemical analysis or standardization: YES	
12-Week treatment period			Placebo group: YES	
			Randomization: YES	
			Double-blind: YES	
			Appropriate statistical analysis: YES	
Single-blind, placebo controlled, parallel study	MRS	Severity of menopausal transition sympt. ↓*	Part of the plant: YES	Fatima and Sultana (2017)
Perimenopausal women	Severity of menopausal transition symptoms	MRS ↓*	Origin: YES	[82]
TT fruit extract			Phytochemical analysis or standardization: NCS	
			Placebo group: YES	
			Randomization: YES	
			Double-blind: NO (single-blind)	
			Appropriate statistical analysis: YES	
Prospective, randomized, double-blind, placebo controlled trial,	FSFI score	FSFI ↑*	Part of the plant: NCS	Vale et al. (2018)
Premenopausal women with diminished libido	QS-F score	QS-F ↑*	Origin: YES	[81]
TT extract	Serum testosterone	Serum testosterone ↑*	Phytochemical analysis or standardization: NCS	
			Placebo group: YES	
			Randomization: YES	
			Double-blind: YES	
			Appropriate statistical analysis: YES	

MF, mount frequency; IF, intromission frequency; ML, mount latency; IL, intromission latency; EL, ejaculation latency; PEI, post-ejaculatory interval; ICP, intracavernous pressure; NCS, not clearly specified; cAMP, adenosine 3′,5′-cyclic monophosphate; cGMP, guanosine 3′,5′-cyclic monophosphate; FSH, follicle-stimulating hormone; LPO, lipid peroxidation; SOD, superoxide dismutase; CAT, catalase; GPx, glutathione peroxidase; GST, glutathione; S, transferase; AR, androgen receptor; IGF-1R, insulin growth factor 1 receptor; UHPLC-Q-TOF/MS, ultra-high performance liquid chromatography-quadrupole-time of flight mass spectrometry; GR, glutathione reductase; GSH, glutathione; 17β-HSD, 17β-hydroxysteroid dehydrogenase; LH, luteinizing hormone; MAP, mean arterial pressure; eNOS, endothelial nitric oxide synthase; urinary T/E ratio, urinary testosterone/epitestosterone (T/E) ratio; CK, creatine kinase; ED, erectile dysfunction; IIEF, International Index of Erectile Function; SQoLM, Sexual quality of life questionnaire male; FSFI, Female Sexual Function Index; GEQ, Global Efficacy Question; MRS, menopause rating scale; QS-F, Sexual Quotient Female Version; *, statistically significant difference as compared with the control/placebo; #, statistically significant difference as compared with the positive control group.

**Table 4:** In vitro and in vivo pharmacological studies and the study design evaluation.

Herbal Drug and Subjects	Assay/Parameters	Outcome of Treated Group	Study Design Evaluation	Reference
In Vitro Studies				
TT fruit extract	α-Glucosidase	Activity inhibition on all tested enzymes	Part of the plant: YES	Lamba et al. (2011)
	Aldose reductase		Origin: YES	[99]
			Phytochemical analysis: NCS	
			Control group: YES	
			Positive control group: YES	
			Appropriate statistical analysis: YES	
TT seeds	α-Amylase	Concentration- inhibition of enzyme activity	Part of the plant: YES	Ponnusamy et al. (2011)
	Kinetic studies.		Origin: YES	[96]
			Phytochemical analysis: YES (identification reactions, GC/MS)	
			Positive control: YES	
			Appropriate statistical analysis: YES	
TT leaves	Lipase	Activity inhibition on all tested enzymes	Part of the plant: YES	Ercan and El (2016)
	α-Amylase		Origin: YES	[95]
	α-Glucosidase		Phytochemical analysis: YES (spectrophotometric)	
			Positive control: YES	
			Appropriate statistical analysis: YES	
In Vivo Animal Studies				
Male Swiss albino rats with STZ-induced diabetes (55 mg/kg)	BW, BG, Hb, HbA1c, TG, TC, HDL, LDL-c	BW ↑*	Part of the plant: YES	El-Tantawy and Hassanin (2007)
TT aerial part extract	Histopathological analysis of the pancreas	BG ↓* after 2,4, and 6 h	Origin: YES	[98]
		HbA1c returned to the normal values	Phytochemical analysis: NO	
		HDL ↑*	Control group: YES	
		TG, TC, LDL-c ↓*	Positive control group: YES	
		Histological structure was less affected as compared with the control group	Appropriate statistical analysis: YES	
Wistar rats with STZ-induced diabetes (50 mg/kg)	BG, BW, HbA1c, INS, GLG	BG ↓*	Part of the plant: YES	Lamba et al. (2011)
TT fruit extract	Urinary albumin levels	BW ↑*	Origin: YES	[99]
		HbA1c, GLG ↑	Phytochemical analysis: NCS	
			Control group: YES	
			Positive control group: YES	
			Appropriate statistical analysis: YES	
Male Wistar rats with STZ-induced diabetes (40 mg/kg)	BG	BG, PT, APTT, TC, TG, LDL, ALT, AST, ALP, glucose-6-phosphatas, fructose-1, 6-bisphosphatase, LPO ↓ *	Control group: YES	Kalailingam et al (2014)
Diosgenin	HbA1c	HDL, SOD, CAT, GSH ↑ *	Positive control group: NO	[101]
	TC, TG, HDL, LDL, AST, ALP		Appropriate statistical analysis: YES	
	PT, APTT			
	Hepatic glucose-6-phosphatase, fructose-1, 6-bisphosphatase SOD, CAT, GSH, LPO			
Male Sprague Dawley rats with type 2 diabetes induced with high-fat diet (HFD) + STZ (35 mg/kg)	BG, INS, BW	BG ↓ *, INS ↑ *, BW ↑ *	Control group: YES	Tharaheswari et al. (2014)
Diosgenin	FFA, TNF-α, IL-6, leptin	FFA, TNF-α, IL-6, leptin ↓ *	Positive control group: NO	[102]
	HOMA-IR, HOMA-B, QUICKI	HOMA-IR, HOMA-B, QUICKI – improved values	Appropriate statistical analysis: YES	
	In tissue homogenate were determined: LPO, GSH, SOD, CAT, GPx	Increased adipose tissue mass		
	Histopathological analysis of pancreas	Enhanced PPARc expression		
	Quantification of adipose PPAR γ	Good interaction of diosgenin with PPAR γ		
Glucose-loaded normal rabbits,	FBG at 30 min, 1, 2, 3 h after dosing	FBG ↓* at 2 hours	Part of the plant: YES	El-Shaibany et al. (2015)
TT aerial parts extract	Acute toxicity study	No toxicity	Origin: YES	[100]
			Phytochemical analysis: YES (TLC)	
			Control group: YES	
			Positive control group: YES	
			Appropriate Statistical analysis: YES	
Male Wistar rats with STZ-induced diabetes (55 mg/kg)	BG	BG ↓*	Part of the plant: YES	Tag et al. (2015)
TT fruit extract	INS	INS ↑*	Origin: YES	[85]
			Phytochemical analysis: YES (identification reactions)	
			Control group: YES	
			Positiv control group: YES	
			Appropriate Statistical analysis: YES	
Sprague Dawley rats with type 2 diabetes induced with high-fat and high-sugar feeding and STZ (30 mg/kg)	BG	BG ↓	Part of the plant: NO	Zhang et al. (2019)
Gross saponins of TT	BW	No significant differences in BW	Origin: YES	[86]
			Phytochemical analysis: NCS	
			Control group: YES	
			Positive control group: YES	
			Appropriate statistical analysis: YES	
Clinical Studies				
100 Patients suffering from DM with microalbuminuria	BG	BG ↓ *	Part of the plant: NCS	Ramteke et al. (2012)
Ayurvedic preparation with TT	BP	BP ↓ *	Origin: NCS	[105]
	Urine albumin	Urine albumin ↓*	Phtochemical analysis or standardization: NO	
			Placebo group: NO	
			Randomization: YES	
			Double-blind: NCS	
			Appropriate statistical analysis: YES	
Double-blind randomized placebo controlled clinical trial	FBG, BG 2-hour postprandial HbA1c	BG ↓*	Part of the plant: NCS	Samani et al. (2016)[104]
Ninety-eight women with diabetes mellitus type 2	TG, TC, LDL, HDL	TC, LDL ↓*	Origin: YES	
TT extract		HbA1c, TG, HDL - no significant differences as compared with the placebo	Phtochemical analysis or standardization: YES	
			Placebo group: YES	
			Randomization: YES	
			Double-blind: YES	
			Appropriate statistical analysis: YES	

NCS, not clearly specified; GC/MS, gas chromatography-mass spectrometry; TLC, thin layer chromatography; STZ, streptozotocin; BW, bodyweight; BG, blood glucose; Hb, hemoglobin; HbA1c, glycosylated hemoglobin; TG, serum triglycerides; TC, total cholesterol; HDL, high density lipoprotein; LDL-c, low density lipoprotein cholesterol; INS, insulin; GLG, glycogen; FBG, fasting blood glucose; AST, aspartate aminotransferase; ALP, alkaline phosphatase; PT, prothrombin time; APTT, activated partial thromboplastin time; SOD, superoxide dismutase; CAT, catalase; GSH, glutathione; LPO, lipid peroxidase; FFA, serum free fatty acids; TNF-α, tumor necrosis factor-α; IL-6, interleukin-6; HOMA-IR, homeostasis model assessment of insulin resistance; HOMA-B, homeostasis model assessment of β-cell function; QUICKI, quantitative insulin sensitivity check index, PPARγ, peroxisome proliferator-activated receptor gamma; GPx, glutathione peroxidase; BP, blood pressure; *, significant difference as compared with the control group and the placebo group.

**Table 5:** Toxicological information of some compounds from the U.S. National Library of Medicine [126].

Compound	Toxicological Information
Diosgenin	Oral LD50 (rat) > 8 g/kg
	Intraperitoneal LD50 (rat) 4872 mg/kg
	Oral LD50 (mouse) > 8 g/kg
	Intraperitoneal LD50 (mouse) 3564 mg/kg
Dioscin	Subcutaneous LD50 (mouse) >300 mg/kg
	Oral TDLo (rat) 1050 mg/kg/1W (intermittent)
	Oral TDLo (mouse):400 mg/kg/10D (intermittent)
Tigogenin	Intraperitoneal LDLo (rat):10 mg/kg
Harmine	Intramuscular TDLo (man):3 mg/kg
	Intravenous LDLo (cat) 10 mg/kg
	Subcutaneous LDLo (frog) 300 mg/kg
	Subcutaneous LD50 (mouse) 243 mg/kg
	Intravenous LDLo (mouse) 50 mg/kg
	Subcutaneous LD50 (rat) 200 mg/kg
Harmane	Intraperitoneal LD50 (mouse) 50 mg/kg
	Interperitoneal TDLo (rat) 1 mg/kg
	Intraperitoneal LD50 (rabbit) 200 mg/kg
Harmaline	Subcutaneous LD50 (rat) 120 mg/kg
	Subcutaneous LD50 (mouse) 120 mg/kg
	Intraperitoneal TDLo (rat) 4 mg/kg
Norharmane	Oral TDLo (rat) 1050 mg/kg/6W (continuous)

LD50, median lethal dose; TDLo, lowest published toxic dose; LDLo, lowest lethal dose.
